# When cultural values meets professional values: a qualitative study of chinese nurses’ attitudes and experiences concerning death

**DOI:** 10.1186/s12904-022-01067-3

**Published:** 2022-10-14

**Authors:** Jiong Tu, Manxuan Shen, Ziying Li

**Affiliations:** 1 School of Sociology and Anthropology, Sun Yat-sen University, Guangzhou, China; 2 Department of Geriatrics, First Affiliated Hospital of Sun Yat-sen University, Guangzhou, China

**Keywords:** Death, Dying, Palliative care, Nursing profession, Qualitative study, China

## Abstract

**Background:**

In China, there is a culture of death-avoidance and death-denying. Influenced by this distinctive socio-cultural views surrounding death, nurses often find it challenging to handle death and care for dying patients. This study explores the nurses’ attitudes and coping strategies concerning death and caring for dying patients in a cultural context of death taboo.

**Methods:**

This research is a qualitative study that employs in-depth, semi-structured interviews with nurses from two major hospitals in Guangzhou, China. Overall, 28 nurses from four departments with high patient death rate were recruited and interviewed. All of the interviews were analyzed thematically.

**Results:**

The nurses who participated in this study expressed attitudes toward death and caring for dying patients from both a personal dimension and a professional dimension. The personal dimension is influenced by traditional culture and societal attitudes towards death and dying, while their professional dimension is congruent with the nursing and palliative care values concerning death and dying. With an obvious discrepancy between these two dimensions, Chinese nurses adopt three strategies in their practice to solve this tension: boundary-drawing to separate their personal and professional life, complying with the existing cultural values at work, and constructing positive meanings for end-of-life care.

**Conclusion:**

In a society that traditionally avoids making any reference to death, it is useful to reduce cultural taboo and construct positive meanings in end-of-life care, death education and the development of palliative care. Meanwhile, nurses also need institutional support, education and training to transition smoothly from a novice to a mature professional when handling patient death.

**Supplementary Information:**

The online version contains supplementary material available at 10.1186/s12904-022-01067-3.

## Introduction

China is aging rapidly with over 190 million people (13.5% of its population) aged above 65 years old by 2020. [1] Nurses play a key role in providing care for older patients and patients with terminal illness. With an aging population, there is an increasing requirement for more nurses to work in the area of taking care of older patients and patients with lifethreatening illnesses. It is thus necessary to explore the nurses’ attitudes toward death and dying, and develop relevant measures to prepare them more effectively to provide care for an aging society.

The current literature covers many aspects of nurses’ attitudes toward death, dying and palliative care. Many researches explore the associations between nurses’ characteristics and their attitudes toward death and caring for dying patients. These researches reveal that nurses’ specialty, age, gender, educational level, years in practice, place of work, religious belief, etc. all have an impact on their attitudes toward death and caring for dying patients. [2–5] Nurses are also influenced by the culture of the society in which they live. [6, 7] Culture provides a distinctive perspective of death as a whole and cultural factors in particular influence nurses’ experiences of patient death. [8].

In China, there exists a culture of death-avoidance and death-denying. [9] Influenced by the distinctive socio-cultural views surrounding death, health professionals often find it challenging to handle death and dying. Compared to American nursing students, Asian nursing students are significantly more afraid of death and more averse to interacting and discussing death with dying patients. [10] For practice nurses in China, many struggle with negative emotions of anger, doubt, fear, and anxiety, feeling uncomfortable in the face of death and dying. [11] In the clinical context, the disclosure of information on death and dying is also a taboo, even information about serious diagnoses and prognoses is often held back from the patients by the family members, [12] which jeopardize effective communication between the health professionals and the dying patients. [9, 13] The end-of-life care in general has been negatively influenced by traditional Chinese culture. [14].

At the same time, nurses’ professional identity can reduce their fear of death, death avoidance and anxiety, and it has been suggested that Chinese nurses should be provided with appropriate death education. [15] Receiving death education before embarking on clinical practice is helpful for nursing interns to care for dying patients. [16] Yet in China, there is a lack of death education in general [17] and a lack of an appropriate death education curriculum for health professionals in particular. Palliative care education is limited and disparate, and even medical teachers hold an ambivalent attitude toward palliative care and its education. [18, 19] Nursing students receive little training on death and palliative care, and possess a low level of knowledge and self-efficacy regarding palliative care. [20, 21] Practice nurses also lack professional knowledge and skills related to caring for terminal patients, and are poorly-prepared to cope with death-related issues. [3, 11, 14] Their competence in areas such as psychological and spiritual care, and ethical and legal issues, is especially low. [22].

Existing studies reveal various factors that influence Chinese nurses’ attitudes and experiences concerning death and dying, yet most of these researches are quantitative in nature.^[[2–5][11][15–17][20–22]]^ There is a lack of qualitative research that explores nurses’ lived experiences and nuanced feelings concerning death and caring for dying patients. Moreover, the current research depicts nurses as passive receivers of their culture, neglecting their agency in actively incorporating different socio-cultural values into their care of dying patients. This study explores nurses’ attitudes and coping strategies concerning death and caring for dying patients within a death-avoidance cultural background.

## Methods

### Aim

To explore the nurses’ attitudes and coping strategies concerning death and caring for dying patients in a cultural context of death taboo.

### Design

A qualitative study with individual semi-structured interviews was conducted between 2020 and 2021. Due to the outbreak of Covid-19 pandemic, the original plan to enter hospital for a long-term ethnography was interrupted. Alternatively, we chose individual interview as the method of data collection. The qualitative study with in-depth semi-structured interviews provides us with a deep, nuanced understanding of the experiences of nurses from their own perspective. [23].

### Data collection and participants

The interviewees were recruited through purposive sampling. Overall twenty-eight registered nurses were intentionally recruited from four departments (Oncology, Neurology, Geriatrics and Respiration) of two major hospitals in Guangzhou, a metropolitan area in southern China. All of the hospitals have no a palliative care department, as most public hospitals in China. In the above four departments, most of the patients were older people or had serious conditions, so these nurses were more likely to be exposed to death and family grieving. Besides, the interviewees were recruited with maximum variation in terms of age and working experiences in order to describe a range of possible experiences and attitudes toward death (see Table 1 below).

The face-to-face interviews were conducted by the researchers (JT, MS, ZL) at the nurses’ workplace or other places of their choice (e.g. lounge or coffee shop near their hospital). Each interview lasted from one to two hours, and was electrically recorded. A semi-structured interview guide was used during the interviews (see Appendix 1). The guide was developed based on the research aim and focused on the following aspects: the first experience of patient death, attitudes toward death and dying, and how to handle the challenges of working in close proximity to death. The semi-structured guide served as a series of “prompts” [24] in the interview for respondents to talk about their own perspectives and attitudes with some freedom. The interviews stopped when the saturation was reached, which means that the data had become repetitive and no new information was emerging from the interviews. [25].

### Data analysis

All interviews were transcribed verbatim and imported into Nvivo 11 for coding. Thematic analysis was used to analyze the data. [26] Two authors (JT, ZL), both familiar with qualitative methods, analyzed the data. They read and re-read the transcripts to familiarize themselves with the data, and then independently coded the transcripts. All of the initial codes were further analyzed to identify themes and subthemes. The two authors then met to compare the themes and had a discussion until they reached agreement about them. Overall, two main themes and six subthemes were identified and defined by the research team (see Table 2). Verbatim quotations were presented below to provide support for each theme.

### Ethical considerations

#### Ethical approval

was granted by the authors’ institution . All of the interviewees were informed about the aim and purpose of this study and agreed to participate in it. The interviewees and their affiliations were anonymized to protect their privacy.

## Results

### Characteristics of the nurses interviewed

All interviewees were female, aged between 24 and 51 years old with an average age of 34. Our interviewees cover both junior and senior nurses with years of practice range from 1 to 33 years (with an average length of 13 years).

**Table 1:** Characteristics of the nurses interviewed (N = 28)

Characteristics	N (%)
**Age group**	
< 30	10(35.7%)
30–39	11(39.3%)
≥ 40	7(25.0%)
**Gender**	
Female	28 (100%)
**Years of practice**	
< 5 years	8(28.6%)
5–10 years	6(21.4%)
≥ 10 years	14(50.0%)
**Position**	
Head nurse	3(10.7%)
Regular nurse	25(89.3%)
**Department**	
Respiration	9(32.2%)
Neurology	4(14.3%)
Oncology	7(25.0%)
Geriatrics	8(28.5%)

### Attitudes toward death and dying: social values vs. professional values

The nurses describe a personal dimension and a professional dimension in their attitudes toward death and caring for dying patients. The personal dimension is influenced by traditional culture and societal attitudes toward death and dying, while the professional dimension is congruent with the nursing and palliative care value concerning death and dying. There is an obvious discrepancy between these two dimensions. While social-cultural beliefs profoundly influenced the nurses’ values, especially during the initial stage of their nursing career, the nurse profession shaped the nurses’ attitudes toward death in particular as they followed their professional trajectory, changing from a novice to a mature professional. Overall, there are three obvious differences between the two dimensions.

#### Death avoidance in daily life vs. death as an unavoidable part of nursing work

Nurses we interviewed report that the traditional death taboo culture affects their attitudes toward death. According to the interviewees, it is distressing to contemplate death. Since death signifies unfortunate event, talking about and encountering death may also bring misfortune and bad luck, most of them avoid death and avoid face-to-face encounters with a corpse in daily life. The culture of avoidance deepens the nurses’ negative feelings about caring for dying patients.

*I’ve always heard about death (in a negative way)… Death means unlucky, bad luck in Chinese culture… I’ve had a fear of death since I was a child. I feel death is a terrifying thing. People even swear by saying “You’re going to die” so, normally, there’s a taboo about death and we’re a little afraid of it… (N2, 25 years old, respiration)*.

*(For the first death encountered) about ten years ago, I didn’t come forward to do post-mortem care…I didn’t dare to. When I knew that a patient was gone, I might stay away as far as possible, not daring to come forward. During my internship, it was (mostly) my teacher who did these things (post-mortem care). I stayed at the back, (trying to) avoid it… Escape, I should say, just escape. (N4, 35 years old, respiration)*

Yet, the frequent occurrence of death in hospital makes it an unavoidable part of nursing work. Faced with death, the interviewees report they must obtain knowledge and develop skills concerning how to provide care for the dying, how to respond when a patient’s health rapidly deteriorates and what to do if a death actually occurs. In practice, nurses have a series of procedures that they must follow before and after a patient dies: monitoring the bodily symptoms and preparing for resuscitation before the patient’s death, informing the family after the death, providing post-mortem care, completing the required documentation, etc. Learning from practice, nurses gradually accept the fact that death is an unavoidable part of their work, and come to grips with it.

*(In the oncology department), basically, there’s at least one (death) a week. Even though I’m not on duty every day, I encounter (patient death) every week. (N20, 41 years old, oncology)*

*I was on night shift. For the whole night, I was worried and on alert. Our colleagues all said he (the patient) couldn’t survive the night. What did I do? I kept monitoring him all night long, and reviewed (the procedure) all the time to see what should be done (if death occurred), prepared everything in advance. Then I found the nurse aid (to request help in advance) and got all the medicine ready (for resuscitation). I didn’t want to appear unprofessional when death really came. Yes, I wrote all of the procedures down on paper and put it on the table… (N27, 25 years old, geriatrics)*.

Seeing death from a medical point of view, the nurses also try to accumulate knowledge and experience from their care of dying patients. When death occurs during their shift, the nurses report experiencing a sense of failure and guilt sometimes, but what they often do afterwards is to review the medical and nursing procedure, to see if any mistakes were made and what should be improved.

*I would think, when I care for my patients tomorrow, should I pay more attention? For some patients, I know they will definitely come to the end (death), so I’m fine (about accepting their death) but, for other patients (who die suddenly), I will learn from the (unfortunate) case, and actively carry sputum aspiration next time, or even if his blood flow is fine, I think I should still be cautious and give him some additional care. (N2, 25 years old, respiration)*

#### Death as a horrible, frightening event vs. death as a natural part of life

Influenced by the social values, nurses we interviewed predominantly have a negative attitude toward death, linking death to a horrible, terrifying image, especially during the early stage of their career. In existing nursing education, death is rarely mentioned. Nurses do not know much about death, dying and end-of-life care before actually practicing nursing. When recalling their initial encounters with death at the start of their career, both junior and senior nurses mention similar experiences of feeling: scared, anxious, shocked, sad, sorry about the loss of a life, and feeling sympathetic with the deceased. These initial death encounters left them with a deep impression and had a significant impact on them in the ensuing period, causing, for instance, bad moods, nightmares, and poor sleep.

*When I had just graduated (from nursing school), the first time I encountered such a case (patient death), I was indeed very afraid. I went in (to the ward) alone. In fact, I was very afraid, and her eyes were not closed (after death). For a long time afterwards, as soon as I fell asleep, the scene would reappear in my mind… For about one or two months, even now, I can remember the girl’s eyes. I felt very scared at that time… (N8, 37 years old, neurology)*.

*We haven’t been taught about death in class before. Before working in the hospital, I didn’t know how a person actually died in the hospital. All I knew was from the media, watching the news, such as someone committing suicide by jumping into a river, jumping off a bridge, or dying in natural disasters. People died fast in these cases but, when I saw some people die slowly in my department, those in a coma over a long time, with all kinds of tubes in the body, I felt it was great suffering, real suffering. It was a shock for me. (N12, 26 years old, respiration)*

The actual nursing care for a dying patient and post-mortem differ greatly from the nurses’ earlier conceptions of death and dying yet, after working in the nursing profession for some time, many nurses felt more confident about facing death. During nursing work, one becomes familiar with the patients and their illness, knows about the progress of their illness and can often anticipate an upcoming death. Death in the medical arena then becomes predictable or familiar for nurses. Although the death of younger patients and sudden deaths are more difficult for nurses to accept, overall, many nurses interviewed display an attitude of not being surprised to see death after working for a certain period of time. Observing the patient’s passage from life to death, they consider death as a natural part of the life cycle, the end of a disease, which is inevitable in most cases.

*If I’m outside the hospital (encountering death), I’ll be afraid for I don’t know how one dies. Here, I know that he died because of this disease, I know what the disease is, saw its progress, have been taking care of him for a long time, then I wouldn’t be so afraid. (N1, 39 years old, respiration)*

*I have been in (nursing) practice for a long time, seeing many deaths and becoming no longer afraid. After seeing many deaths, I got used to it slowly… (Now) I have a very open attitude. I think this (death) is very normal. Everyone will experience it, no matter whether you talk about it or not… It’s a very objective existence, a normal event that everyone will encounter. Everyone will die, no matter if you are rich or poor. (N9, 36 years old, respiration)*

Over time, nearly all of the nurses interviewed saw themselves becoming more open and comfortable in terms of talking and thinking about death, compared to lay people. Yet, according to the nurses interviewed, deaths at work and those in their personal life remained quite different. Even when they accumulate professional knowledge and are no longer scared of death at work, many would still worry and feel fearful when a death occurs in their personal life.

*Death is the law of nature. Everyone will go to that destination, but talking about it in the (work) environment is different from personally experiencing death (in everyday life). Because I’m in the geriatrics department, I don’t feel any surprise or panic (when a death occurs)…but, when my father died, I still felt scared. He died at the age of 60 due to lung problems…He had already been buried when I arrived home. From then on, we were afraid to go to my father’s room. Even after I returned to work (in the city), for a long time, every time I came off the night shift and returned to my dormitory, I always felt that someone was following me…I’m not scared of death (at work), but when it happens in my (personal) life, my fear increases. (N26, 50 years old, geriatrics)*

#### Prolonging life at all costs vs. death with dignity

Many nurses report that, even though they can accept death at work, they struggle to accept the death of their family members; even though their professional view is that unnecessary treatment should be avoided at the end of life, it is another story when it comes to their own family members. As a family member, one should fight death and make every effort to preserve life of their beloved one, in accordance with the value of filial piety and family duty.

*Sometimes, patients die in the hospital, but their family members simply can’t accept the fact. The views of the family members are really different from those of us medical staff. Maybe we nurses think it’s a relief for the patient, but the family can’t accept the death of their family member. Even though they know that the patient has suffered a lot (with continuous treatment), they still can’t accept the departure. (N21, 51 years old, geriatrics)*

*As a nurse, I would avoid unnecessary treatment for myself but, when it comes to my family, I will try my best to save them so, if we see it from the perspective of the medical staff, it’s a choice, but if we put ourselves in the position of the patient’s family and see it from that angle, it’s different. (N6, 36 years old, respiration)*

There is a conflict between the nurses’ views of a good death and the actual medical practice, which emphasizes treatment and resuscitation. It is almost an intuition for the medical professionals to make their best efforts to save lives. The idea of palliative care has been introduced to the Chinese medical field but has yet to gain wide influence and the majority of hospitals lack a palliative care department. Moreover, there is widespread misunderstanding about palliative care among the public, who equate it with abandoning treatment and awaiting death. Influenced by the family’s wishes and the medical principles for saving life, over-treatment during the end-of-life stage is very common and nearly all of the nurses interviewed were critical of it.

*Intubated, resuscitation, lying on the bed with a respirator… We (nurses) wouldn’t accept that (for ourselves), because we see too many patients suffer greatly. They couldn’t make their own decision, but had to accept the decision of their family members… It’s meaningless to prolong life in some cases. It only causes more pain for the patient, but the family wants us to prolong the life, if one or two family members have not yet arrived (at the hospital to say goodbye), they want us to delay the death… (N22, 44 years old, geriatrics)*.

Having seen patients endure prolonged medical treatment and suffer greatly at the end of life, nurses often hold a different opinion about prolonging life. They ponder over what it means to have a ‘dignified death’ or a ‘good’ death, and most agree that it should be a death with minimal pain, involving dying peacefully in one’s sleep, or dying naturally, without any unnecessary medical intervention. Faced with patients’ deaths, the nurses persuade themselves that death is a relief for patients who have been suffering for a long time. They emphasize the quality of life rather than the length of life, and do not support prolonging life at the cost of patients’ dignity. The suffering of their patients also makes nurses think about their own death. Many report wanting a dignified death, without unnecessary life-prolonging measures.

*(A good death is) natural. There isn’t great pain. Some (patients) pass away in their sleep, naturally. There isn’t much pain in sleep. (For me, a good death means) having dignity. It’s best to die of old age, naturally. Don’t do much (treatment) for me. When I come to the end of my life, I hope my family can be with me, don’t carry out excessive medical treatment. (N8, 37 years old, neurology)*

### Coping strategies: boundary-drawing, compliance and meaning-making

As shown above, tension was often experienced by the nurses in terms of their views about death and caring for dying patients. How do the nurses respond to this tension between their personal and professional values? In this section, we identify three strategies that the Chinese nurses adopt to solve the conflicts within their nursing practice: boundary-drawing to separate their personal and professional life, complying with the existing cultural values at work, and constructing positive meanings for end-of-life care.

#### Drawing boundaries between their personal and professional life

Facing the very different experiences and values concerning death at work and in their daily life, many nurses intentionally draw a boundary between their work and the rest of their life. The aim of this boundary is to constrain death within the professional domain, and distance death from their daily life while working in close proximity to death. The nurses try to limit death to the work domain and temporarily forget about it outside work. They indicate that it is necessary to put work aside after returning home, so they can maintain a balance and continue working.

*At the beginning, I just felt great fear (about death). When I first graduated, I felt that, when patients died, it was a very big thing. At that time, during my internship, if a patient died, he’d stay in my mind for a long time. I’d think about it for a few days, even outside work. Especially if I’d helped to try to resuscitate the patient, I’d think about them for a long time. Now, I think about it at work, but put it aside outside work. (N4, 35 years old, respiration)*

*I know, in my profession, some colleagues segregate (death)… for instance, soon after providing end-of-life care and “packing” a corpse, they shower and change their clothes immediately… Another time, I left work with a colleague, and we encountered (the transportation of a deceased patient) in the elevator at the corner. She immediately retreated and refused to get in the elevator…‘What’re you afraid of?’ I asked her. She said it was OK to encounter death when wearing a work uniform, but she had already changed her clothes and was wearing her normal clothes to go back home. The change of clothes meant a change of status. (N17, 29 years old, oncology)*

The nurses would use physical boundaries to solidify their psychological boundaries, setting a clear boundary between the world of work and life. A nurse’s uniform serves as an ideal symbol of this boundary. Many nurses interviewed regard their work uniform as sacred dress that can protect them from evil forces. They avoid encountering death after changing into their normal clothes. As a normal human being, they may fear death in daily life but, as a professional nurse, they have to care for the dying patients. Wearing a uniform means taking on the nurse’s role and being professional. The nurse’s role while in uniform gives them great courage to face death, helping them to treat death as part of their work and handle it from a professional perspective.

*I don’t know why. When I put on my work uniform, I don’t fear death. Even encountering death directly, I won’t fear but, when I take off my work uniform, I dare not go down stairs, (as) the basement is the morgue but, as soon as I put on my work uniform, it seems that something is covering and protecting me, I dare go anywhere, and even handle death directly. (N1, 39 years old, respiration)*

#### Embedding traditional cultural values into everyday nursing practice

Another way in which the nurses react to death is by embedding traditional cultural values into their everyday nursing practice. Many nurses interviewed admit that they are ordinary human beings, who fear death, like everyone else, and often feel emotionally affected by a patient’s death. While admitting their feelings, many of them utilize existing cultural resources to counter the negativity of death and its related social meaning (e.g. bad luck), such as putting apples at the nursing station (meaning safety in Chinese), wearing red clothes or an amulet to ward off bad luck, using objects such as talismans which will bring good fortune, or reciting a few mantras or holy words shortly after providing end-of-life care.

*For a period of time, I was very afraid, and also very superstitious. For example, I must wear red (clothes) on the day of the Spring Festival and then, when there’s a patient in a critical condition, I must wear red clothes (under my uniform) to strengthen my courage (to care for the patient)… (N23, 44 years old, geriatrics)*.

*Here, Cantonese people have a certain custom. That is, when nurses pull out the (venipuncture, gastric, urethra or endotracheal) tubes for (a deceased) patient, (the patient’s family should) give a small red packet to the nurses to ward off bad luck. Our team leader is from Guangdong. She told the patient’s family to prepare a red packet (containing a few Yuan), and then she went to pull out the tubes…The team leader told us (that the money in) the red envelope must be spent immediately, so we bought some lucky candy (candy packed in red) or something to drink. (N10, 24 years old, neurology)*

Besides following these traditional practices, the nurses report there are some “superstitions” that are widely shared among nurses. They mention that death would happen more to a certain bed number for a period of time, and would occur more on special days like the *Qingming* Festival (or “Tomb-sweeping” day); when death occurred if one was on duty, it meant that nurse was in “black” (meaning having bad fortune) and had to wait for the bad fortune (*fengshui*) to move away. Nurses also follow the wider social rule of avoiding saying ‘die’ or ‘death’ directly in case it brings bad luck. They use black humor or metaphors to joke with and warn each other to be more cautious while on duty, thereby countering the psychological stress of facing death.

*Here, we always say somebody “has gone”, or (use the letter) “D” to mean ‘dead’. Putting someone into a body bag (we call) it “packing”, we just wrap it up like this. We don’t directly say that someone’s dead; it’s not a good feeling. Death is only officially mentioned when we change shift, but not usually said in our daily work. We say that someone has “gone down”, to the morgue (in the basement). (N15, 24 years old, oncology)*

*If patients do die on our shift, we say that she (the nurse) is in black (meaning that she is having bad luck). It’s how we joke with each other, using the metaphor that, when a nurse and a doctor work the same shift, they must be prepared to be very busy (with all kinds of unpredictable incidents, including deaths). (N22, 44 years old, geriatrics)*

#### Constructing positive meanings for end-of-life care

Another measure adopted by the nurses is constructing positive meanings for end-of-life care. The nurses suggest that, rather than bringing bad luck, providing care to dying patients brings them blessings and good fortune, as long as the nurses do their best to care for the patients. Accompanying the patients at the last stage of life and ensuring that they experience a peaceful death is a praiseworthy action, which accumulates karmic merits. It will bring fortune for the nurses (such as having a happy marriage or giving birth to healthy babies). Adding positive meanings to end-of-life care actually encourages nurses to deliver high quality care to dying patients, be more sensitive to patients’ needs and combat the fear for death in their nursing work.

*Maybe at the beginning (of our career), we thought it was unlucky to care for a dying patient but, with more years in practice, I think it’s about sending the patients on the last part of the life journey, and thus is a process of accumulating blessings (from the deceased). We respect them, care for them and see them off for their last leg of journey. Now, I’m not afraid. (N24, 31 years old, geriatrics)*

*Many of us joke that, if you want to get pregnant, simply provide end-of-life care. The patient will bless you, if you give them good care at the end, they’ll bless you, and help you realize your wishes… There are stories shared among us. For instance, a nurse helped to shave off a patient’s beard before he died and, soon later, she got pregnant and gave birth to a very healthy baby. That’s to say, it’s actually a blessing. (N28, 50 years old, Geriatrics)*

The nurses adopt the social value of caring for older people as a virtue to transform the death avoidance culture. It then becomes a privilege to accompany patients on the last part of their life. By providing end-of-life care for the dying and preserving their dignity through post-mortem care, the nurses feel that the patients’ suffering is over and that death is actually a relief for both the patients and their carers.

*Initially, I had nightmares, but later I thought it was a relief for the patient. I thought in this way and it comforted me. (N1, 39 years old, respiration)*

*I believe that, as long as I’ve done it with all my heart and made him comfortable, there’s no regret. It’s also a relief for the patient, so he won’t suffer anymore. (N14, 51 years old, oncology)*

## Discussions

### Main findings

**Table 2:** Nurses’ attitudes & coping strategies concerning death and caring for dying patients

Themes	Personal dimension	Professional dimension
**Attitudes**	Death avoidance in daily life	Death as an unavoidable part of nursing work
	Death as a horrible, frightening event	Death as a natural part of life
	Prolonging life at all costs	Death with dignity
**Strategies**	Drawing a boundary between personal and professional life	
	Embedding traditional cultural values into everyday nursing practice	
	Constructing positive meanings for end-of-life care	

In this paper, we explored Chinese nurses’ complex attitudes toward death and caring for the dying. What we found were two seemingly contradictory perspectives: the personal perspective of encountering death and dying in everyday life, and the professional perspective of caring for dying patients in clinical practice. The personal dimension is influenced by the traditional culture and societal attitude that see death as a frightening event to avoid in daily life and often leading family members to try everything to prolong the life of the dying. The professional dimension is congruent with the nursing professional and palliative care value, regarding death as an unavoidable part of nursing work and a natural part of the life cycle, and a good death calls for dignity and a respect for quality of life. As nurses follow their professional trajectory, their attitudes evolve in regard to death and dying, but these two dimensions still overlap and may sometimes come into conflict.

How do the nurses respond to the tension between these two dimensions? The study reveals three strategies that Chinese nurses adopt to solve this conflict: boundary-drawing to separate their personal and professional life, complying with the existing cultural value at work, and constructing positive meanings for end-of-life care. These measures enable nurses to overcome their fear of death while at the same time providing professional care to the dying.

### What this study adds

Current literature on Chinese nurses’ attitudes toward death and caring for dying patients emphases mostly the aspect of social and cultural taboo. [9, 11, 14, 27] Our study adds to current literature by showing a more complex and nuanced picture of nurses’ attitudes, influenced by both the traditional cultural taboo and professional values. Besides, our study further explores nurses’ coping strategies of working in a conflicting culture environment, treating nurses not as passive receivers of their culture, but those who actively incorporate different socio-cultural values into their care of dying patients. Some of the strategies that the Chinese nurses adopted were similar to those found in the existing literature, such as boundary demarcation by changing into work clothes as part of one’s daily work routine, [28] setting boundaries by making light of death, such as making jokes and using humour. [29] Nevertheless, this research adds to the current literature by identifying nurses’ conscious use, by either adoption or transformation, of the existing cultural values. In a society that decrees avoidance of death, it is useful to reduce cultural taboo and construct positive meanings in end-of-life care.

Nevertheless, the use of these individual coping strategies is often limited. For instance, it is easier to accept older people’s death as a relief, while facing the death of younger patients or traumatic deaths are much more challenging. Moreover, even though the nurses emphasize the need for quality of life in a good death, and acknowledge the importance of hospice and palliative services, it is hard to provide these services for patients in practice, since the majority of the hospitals in China lack a palliative care department, and do not provide such services. [30] Patient death is a harsh and demanding reality for nurses, especially new ones with limited practice experience. [31] The above three coping strategies work at the individual level, but nurses still lack formal institutional support to smooth their transition from a novice to a mature professional with regard to handling patient death. The palliative care and death-related training for nursing staff is insufficient in China. [19] The wider social and cultural attitude toward death plays a major role in shaping the nurses’ attitudes about death at the start of their career, which is predominantly negative. [32] Without preparation, it could come as a shock when a patient dies at the beginning of nurses’ careers.

### Implications

The nurses’ attitudes toward death and dying have a critical effect on the application of hospice and palliative care. These attitudes also impact the nurses’ work experiences, as previous research found that nurses with more positive attitudes toward death experience less burnout. [33] Moreover, health professionals can help patients who are facing death and their family members to prepare better for the upcoming death, but this requires them to be prepared to face and handle death first. The findings of this study have important implications for nurse training and practice.

First, it is vital to provide culturally-sensitive death education and training for nurses. Death education should be added to the nursing curriculum to equip nursing students with a clear understanding of death and dying. End-of-life care training also needs to be provided to nurses, especially junior nurses who are new into practice, to improve their knowledge and skills. In accordance with these strategies, culturally-appropriate and culture-sensitive training should be developed to reduce the cultural taboo and help nurses feel better prepared to discuss and handle patient deaths. Intervention framed within a cultural perspective may help to bridge the gaps between professional and social values, as well as strengthen nurses’ cultural awareness and competence in healthcare contexts.

Second, death education should be offered to the general public in the wider society to reduce the cultural taboo of death. There is a general lack of education about death and palliative care among the public. [27, 34] In China and many other countries where death is a taboo, it is useful to make use of appropriate local cultural resources to encourage the public to feel more comfortable about discussing death, such as transforming the fear of death into regarding a dignified death as a blessing.

Third, professional, emotional and societal support should be provided at workplace to enhance nurses’ resilience to death. Nurses who work in close proximity to death need to access more psychological and emotional counseling to cope with death and dying at work. Institutional support can be integrated into professional mentorship or orientation programs at workplace early in a nurse’s career. Moreover, hospice and palliative care should be more widely promoted and developed in face of an aging society. All health professionals should have a better understanding about palliative care, and hospitals should provide relevant services.

### Limitations

All of the interviewees who participated in this study were female nurses, the predominant group in China’s nursing workforce, yet previous study in China found that there exists a substantial gender difference concerning the attitudes toward death among college students. [17] The possible gender difference among nurses’ attitudes toward death and dying warrants further research.

## Conclusion

The nurses who participated in this study expressed their attitudes toward death and caring for dying patients from both a personal and a professional dimension. The personal dimension is influenced by the traditional culture and societal attitude toward death and dying, while the professional dimension is congruent with the nursing and palliative care value toward death and dying. With an obvious discrepancy between these two dimensions, the Chinese nurses adopt three strategies in their practice to solve conflicts: boundary-drawing to separate their personal and professional life, complying with the existing cultural values at work, and constructing positive meanings for end-of-life care. In a society that traditionally avoids death, it is useful to reduce cultural taboo and create positive meanings in end-of-life care, death education and the development of palliative care. Meanwhile, there are limitations to these individual coping strategies. Nurses need a formal institutional support, education and training in order to transition smoothly from a novice to a mature professional in terms of handling patient death.

## Electronic supplementary material

Below is the link to the electronic supplementary material.

Supplementary Material 1

## Data Availability

The datasets generated and analysed during the current study are not publicly available in order to protect the privacy of the interviewees but are available from the corresponding author on reasonable request.

## References

[1] National Bureau of Statistics. The Main Data of the Seventh National Population Census. 2021. http://www.stats.gov.cn/tjsj/zxfb/202105/t20210510_1817176.html..

[2] Wang L, Li C, Zhang Q, Li Y (2018). Clinical nurses’ attitudes towards death and caring for dying patients in China. Int J Palliat Nurs.

[3] Zheng R, Guo Q, Dong F, Gao L (2022). Death Self-efficacy, Attitudes Toward Death and Burnout Among Oncology Nurses: A Multicenter Cross-sectional Study. Cancer Nurs.

[4] Yang FM, Ye ZH, Tang LW, Xiang WL, Yan LJ, Xiang ML (2017). Factors associated with the attitudes of oncology nurses toward hospice care in China. Patient Prefer Adherence.

[5] Wang L, Chen J, Du Y, Wang Z, Li Z, Dong Z (2017). Factors Influencing Chinese Nursing Students’ Attitudes Toward the Care of Dying Patients. J Hospice Palliat Nurs.

[6] Karadag E, Parlar Kilic S, Ugur O, Akyol MA (2019). Attitudes of Nurses in Turkey Toward Care of Dying Individual and the Associated Religious and Cultural Factors. J Relig Health.

[7] Hagelin CL, Melin-Johansson C, Henoch I (2016). Factors influencing attitude toward care of dying patients in first-year nursing students. Int J Palliat Nurs.

[8] Shorey S, André B, Lopez V (2017). The experiences and needs of healthcare professionals facing perinatal death: A scoping review. Int J Nurs Stud.

[9] Dong F, Zheng R, Chen X, Wang Y, Zhou H, Sun R (2016). Caring for dying cancer patients in the Chinese cultural context: A qualitative study from the perspectives of physicians and nurses. Eur J Oncol Nurs.

[10] Kao SF, Lusk B (1997). Attitudes of Asian and American graduate nursing students towards death and dying. Int J Nurs Stud.

[11] Shi H, Shan B, Zheng J (2019). Knowledge and attitudes toward end-of-life care among community health care providers and its influencing factors in China: A cross-sectional study. Med (Baltim).

[12] Yi TW, Deng YT, Chen HP, Zhang J, Liu J, Huang BY, Wang YQ, Jiang Y. The discordance of information needs between cancer patients and their families in China. Patient Educ Couns 2016; 1;99(5):863-9. 10.1016/j.pec.2015.12.022. 10.1016/j.pec.2015.12.022 26763870

[13] Mei X, Tu J (2021). Values, skills, and decision-making: a cultural sociological approach to explaining diagnostic disclosure. Soc Sci Med.

[14] Zheng RS, Guo QH, Dong FQ, Owens RG (2015). Chinese oncology nurses’ experience on caring for dying patients who are on their final days: a qualitative study. Int J Nurs Stud.

[15] Xie L, Li Y, Ge W, Lin Z, Xing B, Miao Q (2021). The relationship between death attitude and professional identity in nursing students from mainland China. Nurse Educ Today.

[16] Xu F, Huang K, Wang Y, Xu Y, Ma L, Cao Y. A Questionnaire Study on the Attitude towards Death of the Nursing Interns in Eight Teaching Hospitals in Jiangsu, China. Biomed Res Int. 2019;2019:3107692. Published 2019 Sep 16. 10.1155/2019/3107692. 10.1155/2019/3107692 PMC6766148 31637256

[17] Wang Y, Tang S, Hu X, Qin C, Khoshnood K, Sun M (2020). Gender Differences in Attitudes Toward Death Among Chinese College Students and the Implications for Death Education Courses. OMEGA - Journal of Death and Dying.

[18] Willemsen AM, Paal P, Zhang S, Mason S, Elsner F (2021). Chinese medical teachers’ cultural attitudes influence palliative care education: a qualitative study. BMC Palliat Care.

[19] Lio J, Ning X, Wu L, Fu L, Sherer R, He L (2018). Exploring Palliative Care Competency Standards for Medical Education in China: A Survey of National Hospice Service Program Providers. J Palliat Med.

[20] Jiang Q, Lu Y, Ying Y, Zhao H (2019). Attitudes and knowledge of undergraduate nursing students about palliative care: An analysis of influencing factors. Nurse Educ Today.

[21] Zhou Y, Li Q, Zhang W (2020). Undergraduate nursing students’ knowledge, attitudes and self-efficacy regarding palliative care in China: A descriptive correlational study. Nurs Open.

[22] Shen Y, Nilmanat K, Promnoi C (2019). Palliative Care Nursing Competence of Chinese Oncology Nurses and Its Related Factors. J Hosp Palliat Nurs.

[23] Crabtree BF, Miller W (1999). Doing qualitative research.

[24] Jiménez TR, Orozco M (2021). Prompts, not questions: Four techniques for crafting better interview protocols. Qual Sociol.

[25] Francis JJ, Johnston M, Robertson C (2010). What is an adequate sample size? Operationalising data saturation for theory-based interview studies. Psychol Health.

[26] Braun V, Clarke E (2006). Using thematic analysis in psychology. Qual Res Psychol.

[27] Zheng R, Guo Q, Chen Z, Ma L, McClement S (2021). An exploration of the challenges for oncology nurses in providing hospice care in mainland China: A qualitative study. Asia Pac J Clin Oncol.

[28] Ekedahl M, Wengström Y (2006). Nurses in cancer care–coping strategies when encountering existential issues. Eur J Oncol Nurs.

[29] Zheng R, Lee SF, Bloomer MJ (2018). How nurses cope with patient death: A systematic review and qualitative meta-synthesis. J Clin Nurs.

[30] Ling M, Wang X, Ma Y, Long Y (2020). A Review of the Current State of Hospice Care in China. Curr Oncol Rep.

[31] Zheng R, Lee SF, Bloomer MJ (2016). How new graduate nurses experience patient death: A systematic review and qualitative meta-synthesis. Int J Nurs Stud.

[32] Bai Q, Zhang Z, Lu X, Shi Y, Liu X, Chan H (2010). Attitudes towards palliative care among patients and health professionals in Henan, China. Prog Palliat Care.

[33] Guo Q, Zheng R (2019). Assessing oncology nurses’ attitudes towards death and the prevalence of burnout: A cross-sectional study. Eur J Oncol Nurs.

[34] Huang QS (2015). A review on problems of China’s hospice care and analysis of possible solutions. Chin Med J.

