# Correction: When cultural values meets professional values: a qualitative study of chinese nurses’ attitudes and experiences concerning death

**DOI:** 10.1186/s12904-022-01095-z

**Published:** 2022-11-11

**Authors:** Jiong Tu, Manxuan Shen, Ziying Li

**Affiliations:** 1 School of Sociology and Anthropology, Sun Yat-sen University, Guangzhou, China; 2 Department of Geriatrics, First Affiliated Hospital of Sun Yat-sen University, Guangzhou, China

**Correction: BMC Palliat Care 21, 181 (2022)**

**https://doi.org/10.1186/s12904-022-01067-3**

Following the publication of the original article [1], the author reported that the equal contributors were not captured for Jiong Tu and Manxuan Shen

This has been corrected in this article and the original article has been updated.
