# Supplementary material for: When cultural values meets professional values: a qualitative study of chinese nurses’ attitudes and experiences concerning death
Source: BMC Palliat Care. 2022 Oct 14;21:181. doi: 10.1186/s12904-022-01067-3 (PMC9561326; doi:10.1186/s12904-022-01067-3)
Supplement: Supplementary file 1 — Supplementary Material 1 [file 12904_2022_1067_MOESM1_ESM.docx]

## Appendix: Interview Guide

**Nurses' attitudes toward death and experiences of caring for dying patients**

1. How do you feel when thinking about death?
2. What is your attitude toward taking care of dying patients?
3. How was your first encounter with death at work? How did you feel about it?
4. Has this experience of handling death at work changed as you have been in nursing for longer? What are the specific changes? What factors have contributed to these changes?
5. What did you do if a patient is about to die (specific procedures and tasks)? Is there any difficulty associated with this work? What did you do to counter the difficulty?
6. If you were faced with the death of a family member or close friend, how would you feel? Is there any difference compared with facing a patient’s death?
7. In your experience and understanding, what is a good death? What kind of death is a good death and what is less good?
8. In general, what do you think of the death quality of patients in your hospitals? What is the overall quality of patient death in China? Why?
9. What efforts do you think the government, hospitals, health professionals, and families, respectively, can make to help patients to have a good death?
10. Can you tell us about your understanding of end-of-life care? What is your attitude towards hospice care? What is your attitude toward palliative care?
11. Is it possible to provide hospice and palliative care at your hospital? Why? If not, what are the difficulties?
12. Is there any difference between the medical staff and ordinary people concerning attitudes toward death?
13. Was there any death education during your nursing education? Is death education currently available at your hospital? Why? What about death education for the general public?
